# Docking in the Dark: Insights into Protein–Protein and Protein–Ligand Blind Docking

**DOI:** 10.3390/ph18121777

**Published:** 2025-11-22

**Authors:** Muhammad Sohaib Roomi, Giulia Culletta, Lisa Longo, Walter Filgueira de Azevedo, Ugo Perricone, Marco Tutone

**Affiliations:** 1 Department of Biological, Chemical and Pharmaceutical Sciences and Technologies (STEBICEF), University of Palermo, Via Archirafi 32, 90123 Palermo, Italy; 2 Biophysics Institute, National Research Council, 90146 Palermo, Italy; 3 Department of Physics, Institute of Exact Sciences, Federal University of Alfenas, Alfenas 37130-001, Brazil; 4 Molecular Informatics Group, Ri.MED Foundation, 90133 Palermo, Italy

**Keywords:** blind docking, protein–protein docking, small molecule-protein docking, ligand-protein docking, blind docking tools

## Abstract

Blind docking predicts binding interactions between two molecular entities without prior knowledge of the binding site. This approach is essential because it explores the entire surface of the receptor to identify potential interaction sites. Blind docking widely works for both protein–protein and ligand–protein interaction studies. In protein–protein blind docking, the method aims to predict the correct orientation and interface of two proteins forming a complex. Protein blind docking is particularly valuable in studying transient interactions, protein–protein recognition, signaling pathways, tentative and significant biomolecular assemblies where structural data is limited. Ligand–protein blind docking discovers potential binding pockets across the entire protein surface. It is frequently applied in early-stage drug discovery, especially for novel or poorly characterized targets. The method helps identify allosteric sites or novel binding regions that are not evident from known structures. Overall, blind docking provides a versatile and powerful tool for studying molecular interactions, enabling discovery even in the absence of detailed structural information. In this scenario, we reported a timeline of attempts to improve this kind of computational approach with ML and hybrid approaches to obtain more reliable predictions. We dedicate two main sections to protein–protein and protein-ligand blind docking, presenting the reliability and caveats for each approach and outlining potential future directions.

## 1. Introduction

Molecular docking plays a crucial role in the discovery of new drugs. Researchers widely accept it as a computational method in structure-based drug design for predicting the binding affinity and conformation of ligands to the target protein structure [1,2,3,4,5,6,7]. Conventional docking methods employ search algorithms and physics-based and pair interactions-based scoring functions to select the optimal ligand pose that can fit into the active binding site of the protein structure. Conventional docking methods assume prior knowledge of the protein’s binding sites. Still, researchers do not always have this information, especially during the initial drug discovery phase or when analyzing poorly characterized proteins [8,9].

### 1.1. Classical Blind Docking

To address the issue in conventional docking methods, the blind docking approach considers the entire surface of the protein as a potential target to identify the ligand binding sites without prior knowledge of the binding pocket, and thus plays a significant role in the identification of allosteric sites, drug repurposing, and target fishing [10].

Blind docking has a significant impact on the initial stage of drug discovery. This approach relies on physics-based or empirical scoring functions; for this reason, it poses significant challenges in terms of low accuracy and high computational costs due to the large search space in proteins. The full search space slows the approach, even for unseen proteins; however, it has the advantage of providing more interpretable outputs based on physical interactions. Blind docking methods do not require a training dataset [8,9,11,12].

### 1.2. Machine Learning (ML)-Based Blind Docking

The incorporation of ML-based approaches in blind docking such as deep learning (DL) models, graph neural networks, has significantly improved docking speed and enhanced the accuracy of binding pockets identification based on patterns learned from large datasets of known complexes. While ML methods offer an effective approach compared to traditional docking methods, they encounter consistency issues, often working well with training datasets; however, they significantly lose accuracy and performance when applied to unfamiliar protein structures. Comparing traditional methods with ML approaches is sometimes considered biased, as researchers often test them on the entire protein structure rather than specific binding sites, making it challenging to evaluate the performance of both methods [12]. Moreover, the advancement of web server computational tools and GPU-accelerated docking has also significantly refined blind docking, along with computational performance [10,13,14,15,16,17,18].

### 1.3. Focus of This Review

**Figure 1:**
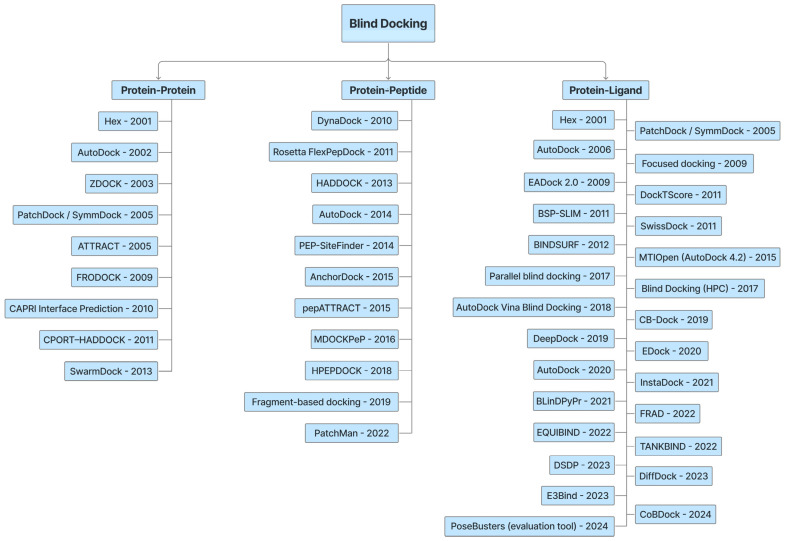
Blind docking tools reported from 2001 to 2025.

**Figure 2:**
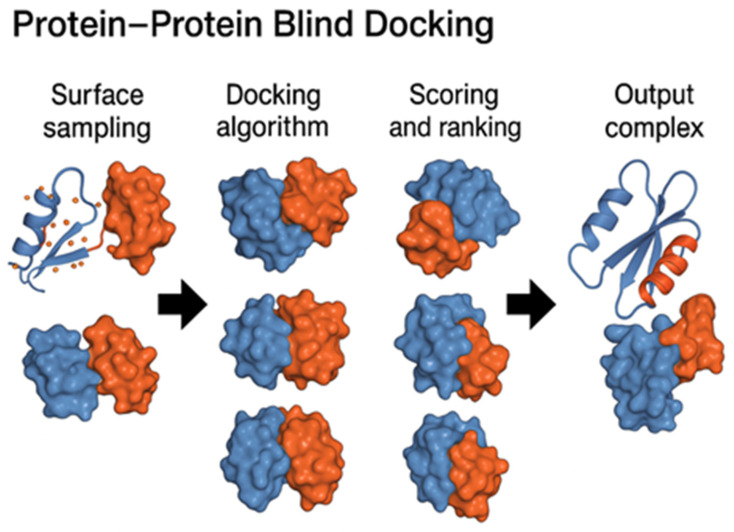
Schematic representation of protein–protein blind docking.

**Figure 3:**
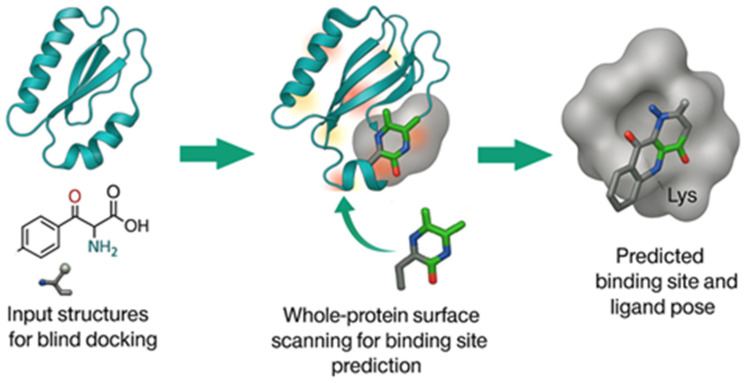
Schematic representation of ligand-protein blind docking.

There have been significant advancements in the development of novel blind docking methods in recent years, particularly for small molecules [17,19,20,21,22,23,24]. This review focuses on published research articles from the last 25 years, highlighting the development of drug discovery methods and tools over a span of more than two decades (Figure 1). We organized the study into two main sections: (1) Protein–protein and Protein–Peptide blind docking, which analyzes the docking methods for proteins or peptides without any prior information of potential binding sites (Figure 2); (2) Small Molecules (Ligands)–Protein blind docking (Figure 3), which follows an approach to dock small molecules inside the multiple protein binding pockets with or without previous knowledge.

We further analyzed the key developments in protein–protein, protein–peptide, and small molecule blind docking, highlighting their comparative evaluation results, integration of novel approaches, methodological innovations, and potential guidelines for future improvement. This review paper also presents valuable insights into the advancements in blind docking over the years, identifying the limitations and advantages of different blind docking methods, comparing traditional, ML, and hybrid approaches, as well as the challenges faced in conventional and ML approaches, which significantly affect consistency, generalizability, computational performance, and accuracy. By discussing all this, we aim to present the refined findings of the drug discovery evolution and its implications for blind docking.

## 2. Protein–Protein and Protein–Peptide Blind Docking

In this section, we report all attempts to develop this computational technique to the best of our knowledge in this century. We begin by discussing the literature regarding all the attempts to predict protein–protein blind docking. The manuscripts are reported in chronological order of their publication, in an effort to understand the evolution of the methods. Table 1 reports working principle, performance, key findings, and limitations to enable readers to compare them and identify similarities and differences among the listed methods.

### 2.1. Protein–Protein Blind Docking

#### 2.1.1. Early Rigid-Body and Geometry-Based Approaches (2001–2005)

The early 2000s marked the foundational phase of protein docking development, characterized by rigid-body, geometry-based methodologies that emphasized computational efficiency over structural flexibility. Hex represented a significant milestone as one of the first programs to employ spherical polar Fourier (SPF) correlation for rapid docking calculations and GPU acceleration, offering unprecedented visualization capabilities at the time [25]. Development of Hex continued until 2013, but no further upgrades were released [26]. Similarly, ZDOCK implemented a fast Fourier transform (FFT)-based scoring function that combined shape complementarity, electrostatics, and desolvation effects, achieving high accuracy across multiple test cases [27]. This new scoring function gave 90% accuracy in 44 test cases. Successively, the authors implemented the algorithm until 2014 as a user-friendly protein docking server, based on the rigid body docking programs ZDOCK and M-ZDOCK to predict structures of protein–protein complexes and symmetric multimers to provide an accessible and intuitive interface [28]. These methods, while computationally innovative, relied heavily on static representations of proteins, treating both receptor and ligand as rigid entities. As a consequence, their predictive power was limited for complexes involving large conformational changes. The introduction of PatchDock and SymmDock (Schneidman-Duhovny et al., 2005) further improved geometric complementarity matching and transformation searches, yet the constraint of structural rigidity persisted [29]. This first generation of docking algorithms thus laid out the computational groundwork for subsequent advancements but fell short in capturing the inherent flexibility of biomolecular interactions.

#### 2.1.2. Transition to Energy-Based and Reduced-Representation Models (2005–2010)

The next stage of methodological evolution sought to overcome rigidity limitations by integrating energy minimization and simplified molecular representations. ATTRACT (Zacharias, 2005) pioneered the use of coarse-grained pseudoatom models and multicopy side-chain strategies to simulate flexible docking more efficiently [30]. This approach enabled exploration of translational and rotational motions, producing near-native predictions for several CAPRI targets and demonstrating that reduced protein models could retain structural accuracy while decreasing computational cost. Concurrently, FRODOCK (Garzon et al., 2009) introduced a hybrid approach combining 3D grid-based potentials with spherical harmonics approximations, improving docking efficiency and scalability across multiprocessor systems [31]. The upgrade FRODOCK 2.0, released in 2016, includes a complementary knowledge-based potential [32]. These innovations signified a methodological shift from purely geometrical alignment to physically informed, energy-driven models. Despite these improvements, both approaches struggled with extensive backbone rearrangements and dynamic interactions, underscoring the need for enhanced modeling of protein flexibility.

**Table 1:** Comparison of different protein–protein blind docking methods in terms of working mechanism, performance, and limitations.

Study (Year)	Docking Method	Working Principle	Performance & Key Findings	Limitations
Ritchie (2003, 2013) [25,26]	Hex	Spherical polar Fourier (SPF) correlation to accelerate calculation	Good results in CAPRI Rounds 1, 2, 3, 5	No more development after 2013
Weng et al. (2003) [27]	ZDOCK	New scoring functions	90% accuracy out of 44 test cases	Not reported
Schneidman-Duhovny et al. (2005) [29]	PatchDock	Connolly complementary patches and transformation	High efficiency for fast transformational search	100 solutions at most
Schneidman-Duhovny et al. (2005) [29]	SymmDock	Like PatchDock, but limited to symmetric cyclic transformation	High efficiency for fast transformational search	100 solutions at most
Zacharias (2005) [30]	ATTRACT	Representation of e-pseudoatoms per residue, multicopy strategy conformational analysis	3 out of 5 CAPRI target RMSD < 1.8 Å	Inaccuracies for extensive backbone conformational changes
Garzon et al. (2009) [31]	FRODOCK	3D grid-based potentials with the efficiency of spherical harmonics approximations	In 4 out of 9 of the CAPRI test cases, the method predicted at least one acceptable solution within the top 10	Slower than PatchDock
de Vries & Bonvin (2011) [33]	CPORT-HADDOCK (Interface prediction + blind docking)	Combined six predictors and docking for refinement	Better than ZDOCK-ZRANK; improved after post-docking analysis	Requires interface prediction first
Torchala et al. (2013) [34]	SwarmDock—CAPRI	176 cases—Generates low energy poses and ranks them	71.6% all poses; 36.4% top 10 poses	Lower accuracy on large proteins
Lensink et al. (2019) [35]	CAPRI round 46	Evaluated automated predictions	High accuracy on easy target; only three models have good quality	Easy and difficult targets create a significant performance gap. Residues in protein binding interfaces were less well-predicted than in previous CAPRi rounds
Harmalkar & Gray (2021) [36]	Comparison of enhanced docking methods	Used MD, Monte Carlo, ML for flexibility	Notable improvement in COVID and Alzheimer targets	Conformational change prediction remains hard
Che et al. (2022) [12]	AutoDock Vina	ML-enhanced blind docking: uses ANN to identify true binding sites	88.6% (top-*n*); 95.6% (top *n* + 2) for LBS (ligand binding site prediction	Still needs improvement in speed

#### 2.1.3. Emergence of Data-Driven and Hybrid Docking Strategies (2010–2015)

Between 2010 and 2015, the field witnessed a paradigm shift toward data-informed hybrid docking approaches that integrated interface prediction, empirical scoring, and post-docking refinement. CPORT–HADDOCK (de Vries & Bonvin, 2011) exemplified this transition by combining consensus-based interface prediction with molecular docking to improve both stability and predictive accuracy [33]. This model demonstrated that docking performance could be substantially enhanced when guided by prior biological information. Similarly, SwarmDock (Torchala et al., 2013) introduced swarm optimization techniques to explore low-energy conformations, achieving competitive results in blind docking scenarios without prior binding-site knowledge [34]. These developments reflected a broader methodological convergence, merging physics-based simulations with heuristic and knowledge-based optimization. The ability to refine docking poses based on predicted interfaces or experimental data represented a significant improvement over earlier blind docking strategies and established a framework for future adaptive methods.

#### 2.1.4. Integration of ML and AI-Enhanced Docking (2016–2022)

The most recent advancements, particularly from 2016 onward, have been driven by the integration of ML and artificial intelligence (AI) to address the persistent challenge of modeling conformational flexibility and binding-induced changes. Studies such as Lensink et al. (2019) highlighted the limitations of existing models in handling complex, conformationally dynamic targets within the CAPRI challenges, reinforcing the need for adaptive computational strategies [35]. Harmalkar and Gray (2021) further emphasized that even with molecular dynamics and Monte Carlo simulations, accurately capturing large-scale backbone movements remains difficult [36]. In this context, Che et al. (2022) demonstrated a significant methodological leap by incorporating artificial neural networks (ANNs) into blind docking workflows, achieving remarkable improvements in ligand-binding site prediction accuracy (88.6% top-*n* and 95.6% top-(*n* + 2)) [12]. The introduction of ML-based models signifies the transition from deterministic, rule-based docking to predictive, learning-driven systems capable of generalizing across diverse targets. These methods represent a new generation of docking algorithms, increasingly capable of autonomously identifying biologically meaningful interactions.

#### 2.1.5. Future Directions and Remaining Challenges

While modern docking methodologies have made substantial progress in predictive accuracy and computational performance, several key challenges remain. Chief among these are the accurate representation of large-scale conformational changes, the dynamic coupling between protein domains, and the computational cost of exhaustive sampling in flexible docking. The next frontier lies in developing integrated hybrid frameworks that combine the physical interpretability of molecular dynamics with the predictive capacity of DL architectures. Enhanced scoring functions informed by experimental data, such as cryo-EM or NMR, and the incorporation of generative AI models for conformational sampling, could further bridge the gap between computational predictions and biological reality. Ultimately, the convergence of physics-based modeling, ML, and high-performance computing is expected to define the future trajectory of docking research, leading to more accurate and biologically relevant predictions essential for structural biology, pharmacology, and rational drug design.

### 2.2. Protein-Peptide Blind Docking

In this subsection, we employed the same workflow, organizing the manuscript in chronological order with the same goals as in the previous subsection. Similarities, differences, working principles, performance, key findings, and limitations are reported in Table 2 to enable readers to compare them and identify similarities and differences among the listed methods. 

The earliest contribution identified in this timeline is DynaDock, introduced by Antes (2010), which represented a foundational step in incorporating peptide flexibility into docking simulations [37]. DynaDock employed a two-step procedure: first scanning the protein–peptide conformational space to identify approximate ligand poses, followed by refinement through optimized potential molecular dynamics (OPMD). This hybrid design—combining sampling and dynamic relaxation—yielded promising results, with best-scoring poses showing peptide RMSD values below 2.0 Å in 11 out of 15 test complexes. The approach effectively bridged rigid docking and molecular dynamics, establishing the conceptual framework for subsequent flexible docking protocols that aimed to capture both conformational variability and binding-site plasticity. A significant advance came shortly thereafter with the introduction of Rosetta FlexPepDock ab initio by Raveh et al. (2011) [38]. Unlike DynaDock, which relied on predefined conformational scanning, Rosetta’s protocol integrated de novo folding of peptides simultaneously with docking, leveraging fragment libraries and coarse-grained structural representations. Its all-atom refinement phase, incorporating complete side-chain modeling for both receptor and ligand, delivered near-native conformations in 25 out of 40 benchmark cases. This marked a transition from heuristic, dynamics-based optimization to a more comprehensive structural search space exploration. Compared to DynaDock, FlexPepDock achieved higher predictive accuracy and more detailed modeling of interfacial side-chain interactions, reflecting a methodological evolution toward physically realistic yet computationally tractable frameworks. In 2013, Trellet et al. further extended the concept of peptide flexibility through an ensemble-based docking approach implemented in HADDOCK [39]. By combining conformational selection and induced-fit mechanisms, the authors demonstrated how an initial peptide ensemble—comprising α-helical, extended, and polyproline-II conformations—could guide the docking process toward biologically relevant binding modes. The results, showing high-quality models for 79.4% of bound/unbound and 69.4% of unbound/unbound cases, clearly outperformed both FlexPepDock and DynaDock, improving interface RMSD values by up to 4.5 Å. The comparative success of HADDOCK illustrated the advantage of sampling diverse conformations early in the docking process and then refining the most promising candidates through flexible molecular adjustments, a principle that would inform much of the subsequent decade’s development in protein–peptide docking. 

In the following years, new algorithms emerged that sought to reduce computational cost while maintaining predictive power. Liang et al. (2014) introduced a two-step functional site identification protocol, where amino acids were screened based on binding energy before assembling dipeptide ligands for docking [40]. This strategy demonstrated a substantial reduction in computational workload while retaining high accuracy, signaling a methodological turn toward efficiency optimization. Similarly, Saladin et al. (2014) developed PEP-SiteFinder, a computational tool capable of scanning entire protein surfaces for potential peptide-binding regions using sequence-based conformational modeling [41]. Its success rate of approximately 90% validated the feasibility of large-scale blind docking, though at the expense of higher computational times. Together, these contributions reflect an emerging emphasis on balancing computational scalability and binding-site accuracy—a theme that continues to shape blind docking research.

Further refinements were achieved with the introduction of AnchorDock (Ben-Shimon et al., 2015) and pepATTRACT (Schindler et al., 2015) [42,43]. AnchorDock advanced blind docking by introducing anchoring site identification and simulated annealing molecular dynamics to handle peptide flexibility, achieving RMSD values ≤ 2.2 Å even without prior structural information. Its design emphasized biological realism by mimicking natural anchoring processes at the protein surface. In contrast, pepATTRACT employed a coarse-grained docking phase followed by atomistic refinement, significantly improving docking speed while maintaining high accuracy—producing correct models for 70% of complexes without any binding-site knowledge. Notably, pepATTRACT’s results were comparable to or exceeded those of FlexPepDock and HADDOCK, marking it as one of the first generalizable blind docking tools suitable for high-throughput applications.

The emergence of MDockPeP by Yan et al. (2016) represented another pivotal advance [44]. This method combined flexible global docking of all-atom peptides with hierarchical refinement, achieving near-native poses in over 90% of both bound and unbound cases when evaluated against the peptiDB benchmark. Its efficiency and accuracy positioned it as a computationally scalable alternative to HADDOCK and FlexPepDock, particularly for large-scale peptide–protein interaction studies. Around the same period, HPEPDOCK (Zhou et al., 2018) offered a hierarchical ensemble-based docking framework that achieved a 33.3% success rate for global blind docking and 72.6% for local docking [16]. Despite a slightly lower accuracy, its computational speed—up to twice as fast as pepATTRACT—made it a practical choice for web-based applications, extending accessibility through its online server.

Subsequent comparative studies, such as that by Agrawal et al. (2019), systematically evaluated six docking methods across 133 protein–peptide complexes [45]. Their findings demonstrated that FRODOCK performed best in blind docking (L-RMSD = 3.72 Å), while ZDOCK excelled in redocking (L-RMSD = 2.88 Å). These results reinforced the importance of combining fast geometric sampling with robust scoring refinement—features exemplified by FRODOCK and ZDOCK—and highlighted the continuing challenge of pose ranking consistency. Around the same time, Balint et al. (2019) introduced Fragment Blind Docking (FBD), which decomposed peptides into smaller fragments to reduce computational complexity while maintaining spatial fidelity [46]. Despite its success in predicting anchoring regions, FBD faced limitations in modeling weaker interactions, emphasizing the ongoing need for improved scoring functions and solvent modeling.

Most recently, PatchMAN by Khramushin et al. (2022) epitomized the modern evolution of blind docking, employing a receptor-centric, motif-based approach rather than relying on peptide sequence data [47]. By identifying structural motifs on the receptor surface to guide docking, PatchMAN achieved 58% accuracy within 2.5 Å RMSD and 84% within 5 Å, outperforming many previous sequence-dependent methods. Its innovation lay in reframing the docking problem from a receptor geometry perspective, effectively addressing the challenge of conformational diversity without explicit peptide folding simulations.

Collectively, these developments illustrate the field’s steady movement toward algorithms that integrate flexibility, efficiency, and structural realism. Early approaches such as DynaDock and FlexPepDock focused on accurate sampling within limited conformational spaces, while later frameworks like pepATTRACT, MDockPeP, and PatchMAN prioritized computational scalability and generalizability. The comparative evolution from dynamics-driven to motif-based methodologies underscores a clear trajectory: from modeling peptide flexibility empirically to predicting peptide–protein recognition through data-informed and geometry-aware frameworks. The next frontier in protein–peptide blind docking will likely involve hybrid AI-assisted approaches that couple deep learning with physical modeling to further refine pose prediction, reduce computational expense, and expand applicability to diverse peptide–protein systems.

**Table 2:** Comparison of different protein–peptide blind docking methods in terms of working mechanism, performance, and limitations.

Study (Year)	Docking Method	Working Principle	Performance & Key Findings	Limitations
Antes (2010) [37]	DynaDock	Two-step algorithm with OPMD (optimized potential molecular dynamics]	11/15 best scoring poses featured a peptide RMSD < 2.0 Å	Time-consuming with respect to the hardware in 2010
Raveh et al. (2011) [38]	Rosetta FexPepDock	Ab initio modeling and coarse-grained representation	18/26 cases in bound form; 7/14 cases in unbound form Perform well on various classes of secondary structure	Computational intensive
Trellet et al. (2013) [39]	HADDOCK	Ensemble, flexible docking	high quality models: 79,4% bound/unbound; 69,4% unbound/unbound 18% better accuracy than FlexPepDock	Not able to model no-helical datasets
Song et al. (2014) [40]	Autodock	Two-step dipeptide blind docking (400 dipeptides),	Dipeptides are used as protein functional site recognizers. Potential role in detecting immunactive sites	limited benchmark on two proteins: human fibroblast growth factor-2 (h-FGF2) and scorpion toxin protein (BmkM1)
Saladin et al. (2014) [41]	PEP-SiteFinder	Scans the full protein surface with peptide conformations	90% accuracy on 41 complexes Creation of the Propensity Index	Long computation time (30–60 min) for each structure. Limited to peptides with a maximum of 30 residues
Ben-Shimon et al. (2015) [42]	AnchorDock	Identifies anchoring spots and uses SA-MD for refinement	RMSD ≤ 2.2 Å; high accuracy (10 out of 13 unbound cases tested)	Relies on anchoring prediction accuracy
Schindler et al. (2015) [43]	pepATTRACT	Coarse-grained + flexible refinement Scans protein surface, then atomistic refinement	70% success without prior site info	Could benefit from ML integration
Yan et al. (2016) [44]	MDOCKPeP	Global docking of all-atom flexible peptide on PeptiDB	95–92.2% success (bound/unbound)	Needs flexibility modeling
Agrawal et al. (2018) [45]	Benchmark study: ZDOCK, FRODOCK, Hex, PatchDock, ATTRACT, and PepATTRACT	Tested six methods on 133 complexes	FRODOCK best (blind); ZDOCK best (re-docking)	Ranking methods need improvement
Zhou et al. (2018) [16]	HPEPDOCK	Peptide flexibility through an ensemble of conformations	33.3% (global); 72.6% (local); 29.8 min runtime	Needs model refinement
Balint et al. (2019) [46]	Fragment-based blind docking	Split peptide and reassembled in complex	Correct placement of anchoring fragments	Simple force fields; no water model C-terminal weakly identified
Khramushin et al. (2022) [47]	PatchMan	Receptor-centric docking using motifs	58% ≤ 2.5 Å; 84% ≤ 5 Å RMSD; 100% sampling	Closed pockets

## 3. Ligand-Protein Blind Docking

In this last section, we continued to discuss the literature evidence in the same way. Table 3 reports the differences, working principle, performance, key findings, and limitations of this approach. The evolution of ligand–protein blind docking from 2001 to 2024 reflects a progressive refinement of computational efficiency, prediction accuracy, and methodological integration. Early approaches, such as Hex [25,26] and PatchDock/SymmDock [29], laid the groundwork for blind docking by exploring geometry-based docking over the entire protein surface. However, these early tools were constrained by rigid-body assumptions and limited energy evaluation functions. Building on these foundations, Hetényi and van der Spoel (2006) introduced one of the first systematic ligand-blind docking approaches using AutoDock, applying it to a set of 43 ligand–protein complexes to evaluate binding selectivity and pose prediction [24]. Their findings demonstrated that blind docking could successfully identify probable binding regions without prior knowledge, providing valuable insight into ligand–protein interactions. Yet, they also highlighted key limitations—such as reduced accuracy in ligand-free or flexible proteins and dependency on the quality of input structures—that continued to shape subsequent methodological improvements.

By 2009, the conceptual boundaries between blind and focused docking began to emerge. Ghersi and Sanchez (2009) proposed focused docking, restricting the search to predicted binding pockets to improve computational efficiency and accuracy compared to fully blind methods [48]. This transition reflected a broader methodological shift toward integrating binding site prediction with docking, emphasizing practical trade-offs between speed and coverage. In parallel, Grosdidier et al. (2009) developed EADock 2.0, which achieved success rates up to 76% in blind docking and even higher in local docking through optimized sampling and scoring strategies [23]. Their results underscored the increasing importance of structure validation and ligand–metal interaction handling—factors that would later inform the design of web-based blind docking platforms.

Between 2011 and 2015, the field experienced substantial diversification, with researchers pursuing different avenues to overcome accuracy and efficiency constraints. Hetényi et al. (2011) systematically compared blind docking and pocket search techniques, demonstrating that both could identify functional binding sites even in challenging protein-ligand systems [49]. Similarly, Lee and Zhang (2011) developed BSP-SLIM, a blind docking method for low-resolution structures integrating I-TASSER predictions with template-based docking, outperforming AutoDock and LIGSITE CSC in RMSD and binding site accuracy [50]. Complementing these developments, Grosdidier et al. (2011) released SWISSDock, a web-accessible implementation of EADock that democratized blind docking by automating setup and validation [51]. The introduction of BINDSURF in 2012 further extended the computational horizon by harnessing GPU parallelism for large-scale screening, improving speed and scalability in multi-site protein systems [52].

The mid-2010s saw a consolidation of blind docking into accessible web-based and high-performance computing (HPC) frameworks. Labbé et al. (2015) developed MTIOpen, integrating AutoDock 4.2 and Vina into a dual-service platform for blind docking and virtual screening [15]. The combination of Diverselib and IPP-lib libraries enabled not only accurate docking but also effective screening for potential PPI inhibitors. By 2017, GPU-accelerated blind docking achieved significant speedups—up to 225× faster than CPU-based implementations—demonstrated by Saadi et al. (2017) [18]. In parallel, Pérez-Sánchez et al. introduced an HPC-based blind docking server [20,21,53], exploring its potential in commercial and pharmaceutical contexts. These studies collectively confirmed that computational acceleration could be achieved without sacrificing predictive fidelity, although improvements in desolvation energy and flexibility modeling remained necessary.

From 2018 to 2020, the field transitioned toward hybrid and automated systems combining traditional algorithms with structural prediction and ML components. Sharmar et al. (2018) emphasized the sensitivity of blind docking to parameter tuning—particularly exhaustiveness—highlighting the need for adaptive control of search parameters [54]. Liu et al. (2019) advanced automation with CB-Dock [17], integrating curvature cavity detection and optimized AutoDock Vina parameters to achieve higher success rates than competing methods such as DockingApp [55], MTiAutoDock [15], rDOCK [56], and SWISSDock [57]. Zhang et al. (2020) introduced Edock, based on Replica-Exchange Monte Carlo (REMC) simulations, achieving superior RMSD and success rates compared to AutoDock Vina and Dock6 [58]. However, Edock’s computational intensity illustrated a recurring challenge: balancing accuracy and resource efficiency, typically taking 2 h per run. In contrast, other tools require only minutes for the same models.

The early 2020s were characterized by the widespread adoption of ML (ML) and DL (DL) to enhance blind docking accuracy and generalizability. Guedes et al. (2021) introduced DockTScore, combining physics-based descriptors with ML models such as SVM and MLR to enhance binding affinity prediction and virtual screening accuracy [11]. At the same time, Mohammad et al. (2021) introduced InstaDock, a GUI-based interface simplifying AutoDock Vina workflows for non-experts [59]. Jofily et al. (2021) followed with BlinDPyPr, integrating cavity-guided and blind docking to combine efficiency with site specificity [9]. Subsequent innovations, such as FRAD (Grasso et al., 2022) [60], introduced MM/GBSA re-scoring to improve energetic evaluation, while DeepDock (Liao et al., 2019) [61] and EQUIBIND (Stärk et al., 2022) [62] applied geometric DL to predict binding poses directly, significantly reducing computational overhead. TANKBind (Lu et al., 2022) further incorporated trigonometric constraints to improve pose accuracy by over 20% compared to previous DL approaches [63]. 

The Integration of ML with physics-based frameworks culminated in a new generation of hybrid models. DSDP (Huang et al., 2023) [10] merged DL-based site prediction with GPU-accelerated sampling, reducing runtime to just over one second per complex while outperforming GNINA and Vina in accuracy. Yu et al. (2023) critically evaluated claims of DL superiority, advocating for hybrid approaches that use DL for site detection and conventional docking for ligand placement [13]. Corso et al. (2023) introduced DiffDock, a diffusion generative model that captured ligand flexibility over non-Euclidean manifolds, outperforming TANKBind and EQUIBIND [64], while Zhang et al. (2022) developed E3Bind, an iterative refinement system inspired by AlphaFold2 that improved accuracy through end-to-end pose optimization [65]. Buttenschoen et al. (2024) later introduced PoseBusters, a comprehensive evaluation framework revealing that, despite progress, DL methods still lag in chemical realism and stereochemical accuracy compared to physics-based tools [66]. Most recently, Ugurlu et al. (2024) proposed CoBDock, a consensus-based method that integrates multiple docking algorithms and cavity detection tools via ML consensus prediction, achieving superior binding site accuracy (0.50–0.88) and RMSD success rates (0.40–0.67) [8].

In 2025, Chen and Zhang investigated whether deep-learning based blind-docking methods can reliably predict allosteric compounds when the ligand binding site is unknown. The authors benchmark several state-of-the-art models (such as DiffDock) on orthosteric and allosteric ligand–protein complexes, demonstrating that a combined workflow (DiffDock followed by local re-docking) can identify both binding mode and binding site for a subset of cases. They show that while these deep-learning methods hold promise for widening the scope of ligand discovery beyond canonical binding sites, current limitations in handling conformationally flexible allosteric pockets and generalizing to unseen targets remain significant barriers [67]. In the same year, two other authors critique the increasingly widespread use of “blind docking” in network pharmacology studies—especially those investigating multi-component systems such as traditional Chinese medicine (TCM). They argue that misuse of blind docking can undermine the validity of network-driven conclusions and provide practical recommendations: avoid blind docking, when possible, validate binding sites via pocket detection tools, report docking parameters explicitly, and where feasible combine docking with molecular dynamics or binding-free-energy calculations [68].

**Figure 4:**
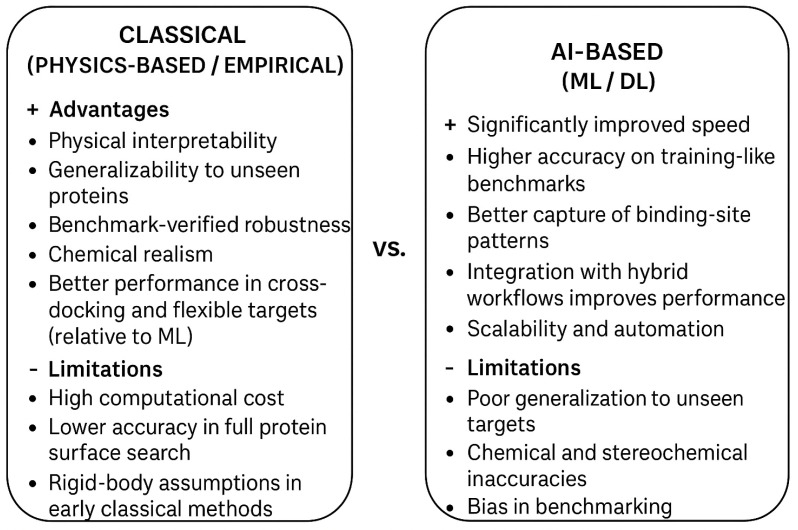
Comparison of Classical and AI-based blind docking methods.

Collectively, the comparative evolution of blind docking from 2001 to 2025 illustrates a clear methodological trajectory—from geometry-based and rigid-body algorithms toward hybrid, ML-driven, and consensus-integrated systems. Early rigid methods emphasized global search at the cost of accuracy, while mid-stage approaches prioritized efficiency through local and focused docking. The latest generation unites data-driven inference with physical realism, enhancing predictive fidelity across diverse protein classes. However, current limitations—such as handling conformational flexibility, integrating solvent dynamics, and ensuring chemical plausibility—underscore the need for continued development of hybrid multi-scale frameworks that unify DL, molecular dynamics, and advanced scoring functions (Figure 4). These integrated systems hold the greatest promise for achieving both speed and accuracy in blind docking across increasingly complex biological environments.

**Table 3:** Comparison of different protein-small molecule blind docking methods in terms of working mechanism, performance, and limitations.

Study (Year)	Docking Method	Working Principle	Performance & Key Findings	Limitations
Ritchie (2003, 2013) [25,26]	Hex	spherical polar Fourier (SPF) correlation to accelerate calculation	Good results in CAPRI Rounds 1, 2, 3, 5	No more development after 2013
Schneidman-Duhovny et al. (2005) [29]	PatchDock	Connolly complementary patches and transformation	High efficiency for fast transformational search	100 solutions at most
Schneidman-Duhovny et al. (2005) [29]	SymmDock	Like PatchDock, but limited to symmetric cyclic transformation	High efficiency for fast transformational search	100 solutions at most
Hetenyi et al. (2006) [24]	Autodock	Drug-sized compounds and proteins up to 1000 residues	Performed well on the system with moderate flexibility	May prove insufficient for systems with a higher degree of induced fit upon ligand binding
Ghersi & Sanchez (2009) [48]	Focused docking	Predict the binding sites, reducing the search space in focused regions	Improved speed and accuracy; useful for reverse screening	Not applicable to global search
Grosdidier et al. (2009) [23]	EADock 2.0	Improved blind and local docking with new seeding and scoring	65–76% (blind), 75–83% (local) success on 260 structures	Sensitive to structure quality; lacks metal interaction handling
Hetényi et al. (2011) [49]	Blind docking + pocket search	Analyzed ligand-free proteins & hydration effect	Performed well on complex cases	Limitations due to multiple pockets
Lee and Zhang (2011) [50]	BSP-SLIM	Template-based blind docking for low-resolution models	RMSD 3.99 Å; better than Autodock and LIGSITE; 25–50% enrichment	Needs improved ligand flexibility modeling
Grosdidier et al. (2011) [51]	SwissDock	Web server based on the engine EADock	251 test complexes: 77% correct Binding Mode	Depends on the number of rotatable bonds of the ligands
Sánchez-Linares et al. (2012) [52]	BINDSURF	GPU-based scan of the whole protein for multiple binding sites	Rapid screening and accurate site prediction for repurposing	Not mentioned
Labbé et al. (2015) [15]	MTIOpen Autodock 4.2	Blind docking and screening via MTiAu-toDock and MTi-OpenScreen	Docked 24/27 proteins accurately; 80% VS ac-curacy	Not mentioned
Saadi et al. (2017) [18]	Parallel blind docking	Used GPU acceleration for large-scale targets	225x/62x faster than CPU; large dataset support	Accuracy of the desolvation energy needs improvement
Pérez-Sánchez et al. (2017) [21] and (2021) [20]	Blind Docking (HPC)	Full surface scanning with HPC; business-oriented	Good industrial potential; positive feedback. Used to identify influenza virus polymerase inhibitors	Data privacy concerns for cloud systems
Sharmar et al. (2018) [54]	AutoDock Vina Blind Docking	Examined exhaustiveness settings on FXa targets	Higher values improved accuracy but reduced speed	Needs parameter optimization and validation
Liu et al. (2019) [17]	CB-Dock	Cavity-based binding site prediction + AutoDock Vina	70% success; better than traditional tools Applied by Ranade et al. (2023) [14] to Dengue Virus protease inhibitors	High computational cost; weak apo performance
Liao et al. (2019) [61]	DeepDock	Universal deep neural network method	Outperform > 4% competing methods	NA
Zhang et al. (2020) [58]	EDock	REMC-based with no prior info or high-res input	RMSD 2.03 Å; better than Dock6/Vina; 67% success	Long run times (~2 h); high resource demand
Guedes et al. (2021) [11]	DockTScore	Scoring functions via ML and physics-based descriptors	Strong results on DUD-E for affinity prediction and VS	Struggles with diverse protein classes
Mohammad et al. (2021) [59]	InstaDock	GUI-based AutoDock Vina (Quick Vina-W) tool	Easy for beginners; large-scale screening is enabled	Lacks ADMET/QSAR, planned for future updates
Jofily et al. (2021) [9]	BLinDPyPr	Combines blind and cavity-guided docking using DOCK6 and FTMap	Achieved 45.2–54.3% pose prediction; 2x faster than traditional DOCK6 blind docking	Needs a GUI/web version; lacks scoring refinement
Grasso et al. (2022) [60]	FRAD	Docking with MM/GBSA re-scoring for pose accuracy	Better performance than traditional docking on >300 complexes	Needs ML integration for larger datasets
Stärk et al. (2022) [62]	EQUIBIND	An SE(3)-equivariant geometric DL model	Better performance than traditional docking methods	Only implicitly models the atom positions of side chains
Lu et al. (2022) [63]	TANKBIND	Trigonometry constraint as a vigorous inductive bias into the model, all possible binding sites	Outperform EQUIBIND, 22% increase in the fraction of prediction below 5 Å; 42% increase with proteins out of the training set	NA
Corso et al. (2023) [64]	DiffDock	Diffusion generative model over the non-Euclidean manifold of ligand poses	Outperform TANKBIND and EQUIBIND with high selective accuracy	NA
Huang et al. (2023) [10]	DSDP	ML-based site prediction + AutoDock Vina pose sampling	29.8% top-1 success rate (1.2 s/run); 57.2% (DUD-E), 41.8% (PDBBind) success rates	Needs improved scoring functions
Yu et al. (2023) [13]	Hybrid: DL + Traditional	DL for site prediction, traditional for ligand docking	DL excels in site prediction; traditional docking is better for pose accuracy	Blind docking alone is unreliable; a hybrid suggested
Zhang et al. (2022) [65]	E3Bind	Equivariant DL model refining ligand pose iteratively	Outperforms traditional and DL tools in docking accuracy	High computational cost; needs diverse datasets
Buttenschoen et al. (2024) [66]	PoseBusters (evaluation tool)	Evaluates docking poses using chemical/physical plausibility	Found conventional tools outperform DL methods on physical accuracy	DL methods fail to match physical realism despite low RMSD
Ugurlu et al. (2024) [8]	CoBDock	Consensus-based blind docking using multi-tool + ML pipeline	Binding site prediction accuracy 0.50–0.88; pose RMSD < 2 Å in 40–67% cases; outperforms other tools	Modular improvements needed for large-scale use

## 4. Conclusions and Future Perspective

### 4.1. Advances

Over the past two decades, blind docking has evolved from rigid-body, geometry-based algorithms to data-driven and hybrid AI-enhanced frameworks that combine physical interpretability with predictive power. Early approaches such as Hex, ZDOCK, and PatchDock provided the computational foundation for large-scale docking by emphasizing geometric complementarity and speed, though they lacked flexibility and struggled to model dynamic protein behavior. The subsequent integration of energy-based potentials and reduced-representation models—exemplified by ATTRACT and FRODOCK—marked a transition toward physically informed methods that improved accuracy at manageable computational costs. Later, hybrid tools such as HADDOCK, SwarmDock, and pepATTRACT further advanced predictive performance by combining empirical scoring with interface prediction and post-docking refinement, effectively bridging the gap between blind and guided docking.

In the realm of protein–peptide docking, the methodological focus shifted toward capturing conformational flexibility and biological realism. Frameworks such as DynaDock, FlexPepDock, and HADDOCK introduced ensemble and dynamic-based simulations that more accurately represented peptide adaptability. Subsequent innovations, including pepATTRACT, MDockPeP, and PatchMAN, demonstrated how coarse-grained modeling, hierarchical refinement, and motif-based recognition could collectively enhance both computational efficiency and structural fidelity. This progression underscores a clear methodological trajectory—from empirical modeling of flexibility to predictive frameworks capable of generalizing across diverse peptide–protein systems.

For ligand–protein blind docking, the field’s development mirrors a steady convergence between physics-based models and ML (ML) architectures. The initial reliance on search algorithms and scoring functions, as seen in AutoDock and EADock, gradually gave way to hybrid frameworks that integrate cavity detection, ensemble sampling, and statistical learning, such as CB-Dock, DockTScore, and EDock. The recent advent of DL (DL) and generative models—including DeepDock, EQUIBIND, TANKBind, DiffDock, and E3Bind—has further accelerated docking by predicting poses and binding sites with reduced computational cost. However, recent benchmark analyses (e.g., PoseBusters [66]) reveal that DL methods, while fast and scalable, still fall short in chemical realism and stereochemical accuracy compared to traditional physics-based approaches. The emergence of consensus and hybrid systems such as CoBDock [8] demonstrates the growing recognition that the optimal path forward lies in integrating the interpretability of physical modeling with the adaptability of AI-driven prediction.

### 4.2. Challenges

Despite these remarkable advancements, several fundamental challenges persist. Chief among them are the accurate modeling of conformational flexibility, solvent and entropic effects, and the reliable scoring of induced-fit and transient interactions. The inherent trade-off between accuracy and computational cost remains a critical constraint, particularly for large or poorly characterized biomolecular systems. Furthermore, reproducibility and benchmark standardization remain open issues, especially in ML-based blind docking, where model generalization beyond training datasets can vary substantially. Different docking studies use heterogeneous datasets and metrics, complicating fair performance comparison. In this scenario, we do not critique data diversity or representativeness of benchmark datasets due to the complexity of this point. ML-based docking models may suffer from overfitting, performing well on training data but poorly on unseen complexes. Additionally, while most studies evaluate binding within native complexes, performance on cross-docking or off-target binding—critical for assessing model robustness and real-world applicability—remains underexplored. Addressing these issues through standardized datasets, transparent reporting, and hybrid benchmarking frameworks will be essential for advancing the reliability and generalization of future blind docking methodologies.

### 4.3. Future Directions

Future progress will likely depend on the development of multi-scale hybrid frameworks that couple molecular dynamics simulations, DL, and generative AI for enhanced conformational sampling and scoring. The incorporation of experimental data—such as cryo-EM densities, NMR restraints, and high-throughput binding assays—into training and validation pipelines will further improve biological relevance and interpretability. Additionally, the integration of GPU-accelerated computation and cloud-based web servers will continue to democratize blind docking, enabling large-scale virtual screening across proteomes and chemical libraries. Ultimately, the convergence of physics-based principles, data-driven learning, and computational efficiency will define the next generation of blind docking, fostering more reliable and biologically meaningful predictions for drug discovery and structural biology, taking into account that misusing blind docking weakens the reliability of findings; therefore, researchers should validate binding sites, clearly report docking parameters, and, when possible, use molecular dynamics or binding-free-energy calculations instead of blind docking.

## Data Availability

No new data were created or analyzed in this study. Data sharing is not applicable to this article.
